# A cross-sectional national survey of community pharmacy staff: Knowledge and antibiotic provision

**DOI:** 10.1371/journal.pone.0215484

**Published:** 2019-04-25

**Authors:** Shukry Zawahir, Sarath Lekamwasam, Parisa Aslani

**Affiliations:** 1 The University of Sydney School of Pharmacy, Sydney, NSW, Australia; 2 Population Health Research Centre, Department of Medicine, Faculty of Medicine, University of Ruhuna, Galle, Sri Lanka; University of Campania, ITALY

## Abstract

**Background:**

Pharmacists’ knowledge about the clinical and legal aspects of antibiotic supply has an impact on appropriate dispensing practice. There are limited studies evaluating community pharmacists’ knowledge of antibiotic dispensing in low and middle-income countries, including Sri Lanka. We aimed (i) to evaluate community pharmacy staff’s self-reported knowledge about antibiotics and dispensing behaviour of antibiotics without a prescription, and (ii) to identify possible factors impacting their antibiotic dispensing behaviour.

**Methods:**

A cross-sectional survey was conducted among a random sample (n = 369) of community pharmacies across all nine provinces in Sri Lanka using a self-administered questionnaire on their antibiotic knowledge and dispensing practice. Data were analysed using descriptive and inferential statistics including; t-test, one-way ANOVA or chi-square test, and binary and multiple logistic regression.

**Results:**

A total of 265 pharmacy staff (210 (79%) pharmacists and 55 (21%) assistants) responded. Overall mean antibiotic knowledge score was 26.1 (SD 3.9; range 1–33, max possible score 34). The overall mean knowledge score t(263) = 2.41, *p* = 0.017, specific knowledge about antibiotic resistance (ABR) t(262) = 4.98, *p* = 0.021 and legal aspects of antibiotic dispensing χ^2^(1, N = 265) = 8.55, *p* = 0.003) were significantly higher among pharmacists than assistants. One in every three pharmacy staff reported that they dispensed antibiotics without a prescription on patient request; however the proportion was close to half when the patient was known to them. About 30% of the staff reported to have supplied antibiotics for minor infections in the week prior to the survey. However, there was no significant difference in the supply between pharmacists and assistants except for acute sore throat (12% vs 23%, respectively; *p* = 0.040). Those pharmacists with higher ABR knowledge were less likely to give out antibiotics without a prescription for viral infections in adults (Adj. OR = 0.73, 95% CI: 0.55–0.96; *p* = 0.027) and children (Adj. OR = 0.55, 95% CI: 0.38–0.80; *p* = 0.002). Awareness of legal aspects of antibiotic supply reduced overall dispensing (Adj. OR = 0.47, 95% CI: 0.30–0.75; *p* = 0.001), and specifically for bacterial infections in adults (Adj. OR = 0.45, 95% CI: 0.20–0.99; *p* = 0.047). Knowledge about antibiotic use and misuse reduced the likelihood of illegal dispensing for common cold (Adj. OR = 0.75, 95% CI: 0.60–0.94; *p* = 0.011) and acute diarrhoea (Adj. OR = 0.76, 95% CI: 0.58–0.99; *p* = 0.048).

**Conclusion:**

Despite the law prohibiting provision, antibiotic dispensing without a prescription continues in community pharmacies in Sri Lanka. Appropriate antibiotic dispensing was associated with high levels of pharmacists’ legal and clinical knowledge about antibiotics. Strategies to change the current practice are urgently needed.

## Introduction

Antibiotic resistance (ABR) is growing at an alarming rate and poses a major threat to the clinical efficacy of antibiotics [1]. ABR increases healthcare costs, length of hospital stay, and morbidity and mortality, in both developed and developing countries [2].

ABR is an inescapable consequence of antibiotic use [3], but it increases hugely with misuse and abuse [4, 5]. Self-medication with antibiotics is one of the major factors contributing to misuse in developing countries [6, 7]. Community pharmacies are primarily responsible for self-medication with antibiotics in low and middle-income countries (LMICs) [4, 6, 8] where infectious diseases are a major burden [6]. Despite the restrictions for the use of systemic antibiotics and recommendations that such medicines be exclusively used under medical prescription, the dispensing of antibiotics without a prescription is still a common practice in this region. Over 50% of all outpatient antibiotics dispensed in most parts of the world are not prescribed by physicians [6, 7, 9]. In Sri Lanka, recent simulated client studies show that 61% of pharmacies dispensed antibiotics illegally when requested by product name [10], and 41% on symptoms-based antibiotic requests and most were supplied inappropriately for minor viral infections [11]. This poor dispensing practices seen in LMICs are attributed to the low level of adherence to the existing laws and regulations on good dispensing practice [10, 12], lack of knowledge and professionalism among pharmacists [12], demand from consumers [10, 12], profit orientation and lack of professionally qualified pharmacists [10, 12]. The lack of knowledge about antibiotics, ABR, and legal aspects of antibiotic dispensing contribute to illegal and inappropriate antibiotic dispensing practice in LMICs [12–14]. Therefore, community pharmacists’ knowledge about antibiotics, ABR, and regulation of supply have a significant impact on their dispensing practice. The factors influencing antibiotic dispensing without a prescription may differ in various developing and developed countries, and attempts in developing interventions need to address the context and focus on the root causes specific to the part of the world.

Although knowledge and professional competency of community pharmacists in antibiotic dispensing are vital to ensure appropriate antibiotic supply to the community, limited studies (and none in Sri Lanka) have evaluated these characteristics in LMICs where antibiotic supply without a prescription is high. Therefore, this study aimed to investigate the antibiotic dispensing behaviour of Sri Lankan community pharmacies. A national survey was conducted to (i) evaluate community pharmacy staff’s self-reported antibiotics knowledge and dispensing behaviour (without a prescription), and (ii) identify possible factors impacting such antibiotic dispensing behaviour.

## Methods

A cross-sectional study was conducted using a validated, self-administered and structured questionnaire among community pharmacy staffs throughout Sri Lanka between December 2016 and September 2017.

### Study population and settings

A systematic sampling of community pharmacies in Sri Lanka, comprising of all types of community pharmacies in the country, including semi-government (Rajya Osusala) and private pharmacies (single, chain and pharmacies in private hospitals) covering all nine provinces of the country was conducted. The study sample was achieved using a proportionate sampling technique in each province, as described below and elsewhere [10].

### Sample size calculation and sampling technique

The original sample size (n = 369) was estimated using standard error of proportions equation with a 95% confidence interval using margin of random error of ±5% based on the results of a previous pilot study conducted in Sri Lanka measuring knowledge about antibiotics among community pharmacists. Sample size estimation has already been reported in detail elsewhere [10]. The response rate in a similar survey conducted among community pharmacists in Portugal was 65% [15] therefore, an additional 30% was added to the original estimated sample size to address non-response rate.

Pharmacies for this survey were randomly selected from all nine provinces of the country (sampling frame). A simple random sampling technique was used in the sample selection. The proportionate sample of pharmacies for each province was derived from the list of pharmacies from the Pharmacy Directory maintained by the National Medicine Regulatory Authority (NMRA), which includes all pharmacies that had legal permission to function (as at April 2016) [16]. A random point in the road map in each province was taken into account to select the first pharmacy on that road as the index pharmacy. Pharmacies were approached throughout the province until the quota was filled. A similar approach was continued in all other provinces [10].

### Data collection

The selected community pharmacies in each province were visited by trained research assistants who were either pharmacy undergraduates or recent graduates.

The questionnaire was distributed to either consented community pharmacist or pharmacy assistant (in the absence of a pharmacist at the time of the visit) in their preferred language (Sinhala, Tamil or English). The most senior pharmacy assistant was approached in the latter case. Community pharmacy staff either completed the questionnaire on the spot or asked the research assistant to collect it at a mutually agreed time and a day. A reminder was made over the phone a day before the agreed collection day. If the questionnaire was not ready on the agreed day, respondents were given an addressed envelope with the postal charges paid to post it to us after filling.

### Ethics statement

This study was approved by the Ethics Review Committee, Faculty of Medicine, University of Ruhuna, Sri Lanka (Reference number 16.11.2016:3.1). Informed written consent was obtained from all participants for the self-reported survey after explaining about the study and its objectives. The consenting pharmacy staff were asked to complete the structured questionnaire described below. The questionnaire was anonymous with no personal identifiable information collected.

### Survey instrument development

The structured questionnaire (S1 Appendix) used in this study was developed through an extensive review of the literature [17–19], pertaining to knowledge, attitudes and practices of community pharmacy staff regarding antibiotics.

The face and content validity of the questionnaire was assessed using a panel of six experts from Australia and Sri Lanka, including two researchers, a pharmacologist, a clinical psychologist, a clinician and a pharmacist. The reliability, clarity, comprehension and time of completion of the modified questionnaire were assessed among ten purposely selected community pharmacists and who were excluded from the final survey. The initial questionnaire was developed in English and translated into the two primary languages spoken in Sri Lanka using forward-backwards and reconciliation process [13, 20].

### Measures

- Socio-demographic and professional characteristics; including age, gender, geographical location, level of pharmacy education, years of working experience in the community pharmacy settings, employment type, employment status, type of pharmacy, and number of pharmacists and pharmacy assistants working at a given time in the pharmacy, were included.
- Knowledge was assessed using four sub-groups (concepts) including knowledge about antibiotics, ABR, use/misuse of antibiotics and legal aspects of antibiotics dispensing. Each sub-group had statements to measure the concept with “Yes”, “No” and “Unsure” response options. Knowledge about antibiotics was measured using three statements. For each statement a score of one was assigned to a correct response, and these scores were summed to get an overall score (0–3). Since the index score of this variable was normally distributed, this variable was treated as a continuous variable to predict antibiotic dispensing behaviours of pharmacists [20, 21]. Knowledge about ABR (12 items), use/ misuse of antibiotics (14 items) and legal aspects of antibiotic dispensing (5 items) were similarly scored and treated as predictors for antibiotic dispensing behaviour. The distributions of the index scores of all the created score variables were normally distributed except legal aspect of antibiotic dispensing. This variable remained highly skewed even in different possible transformed status. Therefore, the six-point index score was re-coded into two levels by a median split (median = 5), poor knowledge about legal aspects of antibiotic dispensing (score ≤4), and high knowledge (score = 5), to use in subsequent analyses.
- Antibiotic dispensing behaviour was assessed using the following six statements: (1) I dispense antibiotics without a prescription if a patient requests it; (2) I give antibiotics without a prescription for a child with minor viral infections; (3) I give antibiotics without a prescription for an adult with minor viral infections; (4) I give antibiotics without a prescription for a child with minor bacterial infections; (5) I give antibiotics without a prescription for an adult with minor bacterial infections; (6) If I know the patient, I dispense antibiotic without a prescription on patient’s request. Each of these items had five response options on a Likert scale: (1) never, (2) some time, (3) half of the time, (4) most of the time, and (5) always.
  Additionally, self-reported rates of antibiotic supply without a prescription in the previous week by pharmacy staff for specific minor infections; including acute sore throat, common cold, wound infections, UTI and diarrhoea were also assessed using a Likert scale with six points (0% = 1 never, 2 = 25% of instances, 3 = 50% of instances, 4 = 75% of the instances, 5 = always and don’t know).
- Pharmacist: Those who reported possessing a degree in pharmacy, proficiency or diploma in Pharmacy, efficiency (apprentice pharmacy training) qualification were categorised as pharmacists and the rest were considered as pharmacy assistants (Table 1).

**Table 1:** Socio-demographic and professional characteristics.

Characteristics	Frequency (%)			
	Overall N = 265	Pharmacists n = 210	Pharmacy assistants n = 55	χ^2^ *p* Value
**Gender**				.931
Male	172 (64.9)	137 (65.2)	35 (63.6)	
Female	91 (34.3)	73 (34.8)	18 (32.7)	
MD	2 (0.8)	0	2 (3.6)	
**Age groups (Years)**				.913
20–29	43 (16.2)	31 (14.8)	12 (21.8)	
30–39	103 (38.9)	85 (40.5)	18 (32.7)	
40–49	65 (24.5)	50 (23.8)	15 (27.3)	
≥50	50 (18.9)	43 (20.5)	7 (12.7)	
MD	4 (1.5)	1 (0.5)	3 (5.5)	
**Geographical area**				.353
Urban	189 (71.3)	147 (70.0)	42 (76.4)	
Rural	76 (28.7)	63 (30.0)	13 (23.6)	
**Level of Pharmacy Education**				N/A
Proficiency^a^	65 (24.5)	65 (31.0)	N/A	
Efficiency^b^	140 (52.8)	140 (66.7)	N/A	
Degree^c^	5 (1.9)	5 (2.4)	N/A	
Pharmacy trainee^d^	11 (4.2)	N/A	11 (20.0)	
No pharmacy education	40 (15.0)	N/A	40 (71.7)	
MD	4 (1.5)	0	4 (7.3)	
**Years of work experience in the community pharmacy**				< .001
≤1	21 (7.9)	8 (3.8)	13 (23.6)	
2–3	27 (10.2)	25 (11.9)	2 (3.6)	
4–5	34 (12.8)	30 (14.3)	4 (7.3)	
>5	178 (67.2)	144 (68.6)	34 (61.8)	
MD	5 (1.9)	3 (1.4)	2 (3.6)	
**Employment type**				.332
Owner (pharmacist or non-pharmacists)	101 (38.1)	84 (40.0)	17 (30.9)	
Employee	161 (60.8)	126 (60.0)	35 (63.6)	
MD	3 (1.1)	0	3 (5.5)	
**Employment status**				.823
Full time	219 (82.6)	175 (83.3)	44 (80.0)	
Part time	43 (16.2)	35 (16.7)	8 (14.5)	
MD	3 (1.1)	0	3 (5.5)	
**Type of pharmacy**				.214
Rajya Osusala (Semi Government)	20 (7.5)	18 (8.6)	2 (3.6)	
Private chain pharmacy	116 (43.8)	88 (41.9)	28 (50.9)	
Single private pharmacy	118 (44.5)	97 (46.2)	21 (38.2)	
Pharmacies in Private hospitals	11 (4.2)	7 (3.3)	4 (7.3)	
**Total number of registered pharmacists working in the pharmacy**				.651
None	7 (2.6)	5 (2.4)	2 (3.6)	
1	179 (67.5)	143 (68.1)	36 (65.5)	
≥2	72 (27.3)	59 (27.3)	13 (23.6)	
MD	7 (2.6)	3 (1.4)	4 (7.3)	
**Number of Pharmacist at any given time**				.530
0	7 (2.6)	4 (1.9)	3 (5.5)	
1	195 (73.6)	158 (75.2)	37 (67.3)	
2	23 (8.7)	18 (8.6)	5 (9.1)	
>2	15 (5.8)	15 (7.3)	0	
MD	25 (9.4)	15 (7.1)	10 (18.2)	

MD–Missing data; N/A–Not applicable

^a^ Pharmacists with two years certificate or diploma qualification including 6 months hospital training

^b^ Pharmacists with an apprentice training program under a trained pharmacist’s supervision

^c^ Pharmacists with B.Pharm or BSc pharmacy qualification

^d^ Individual registered for apprentice pharmacy program and undergoing in the training program

v. Definition of antibiotics: This item was created based on responses of two statements asked from the respondents: (1) Antibiotic is an agent used to kill or inhibit the growth of microorganisms (bacteria, fungus, virus, parasites). (2) Antibiotic is an agent to kill or inhibit the growth of bacteria. Both statements had three response options: (1) Yes, (2) No, (3) Unsure. Respondents were considered having knowledge about the definition of antibiotic if they gave the correct responses to both statements.

### Data analysis

Completed questionnaires were coded, reviewed for accuracy, entered into a database in the SPSS (Version 24.0; IBM Corporation, Somers, NY), and analysed using descriptive and inferential statistics. All categorical variables, including respondents’ socio-demographic and professional characteristics and antibiotic dispensing practices, were expressed as frequencies and percentages. The knowledge scores were calculated as the mean (±SD) or frequency (%). The influence of respondents’ professional and demographic factors on knowledge were tested using t-test, one-way ANOVA or chi-square test. Binary logistic regressions and multiple logistic regressions were performed to examine the association of knowledge about antibiotics, and socio-demographic and professional characteristics on reported antibiotic dispensing practice (dispensing antibiotics without a prescription in general and for specific infections).

All the predictors adjusted in the multiple regression models were entered simultaneously to predict the unique contribution of the variables to the outcomes of interest. The predictors adjusted in the models including gender (male  =  1; female  =  2), geographical area (urban = 1, rural = 2), age (continuous, in years), years of community pharmacy experience (continuous), employment type (owner = 1, employee = 2), employment status (fulltime = 1, part time = 2), knowledge about antibiotics (continuous, score range 0–3), knowledge about ABR (continuous, score range 0–12), knowledge about antibiotic use/ misuse (continuous, score range 0–14) and knowledge about legal aspect of antibiotic use (score 0–4 low knowledge = 0, score 5 high knowledge = 1) were entered into the models.

Odds ratios (OR), adjusted odds ratios (Adj. OR) and 95% confidence intervals (CI) were calculated where appropriate. All *p*-values presented are two tailed and level of significance was set at *p* < .05.

## Results

In total, 267 pharmacy staff (pharmacists and pharmacy assistants) were surveyed (response rate = 72% of the original sample) and 265 participants were included in the final analyses as two questionnaires were incomplete. Of the participants included in this study, 21% (n = 55) were categorised as pharmacy assistants and 79% (n = 210) as pharmacists.

### Socio-demographic and professional characteristics of the respondents

Table 1 presents the difference in sample characteristics of pharmacists and pharmacy assistants. There was no significant difference observed between pharmacists and pharmacy assistants with regards to the socio-demographic and professional characteristics measured except the years of working experience. Pharmacists had worked for significantly longer period than assistants (χ^2^ (3, N = 265) = 26.85, *p* < 0.001). About two-thirds of pharmacy staff were male (172/265, 65%), aged between 30–49 years (168/265, 63.4%) and with more than five years of community pharmacy work experience (178/265, 67.2%). More than two-thirds of community pharmacies were in urban (71.3%) areas. Only about 2% of community pharmacists had a formal university degree in pharmacy and over half of the (53%) pharmacists had an efficiency (apprentice) pharmacy training.

### Self-reported knowledge about antibiotics

Although all mean knowledge scores were numerically higher for the pharmacists compared to the assistants (Table 2), only the mean knowledge score related to ABR was statistically significant t(262) = 4.98, *p* = 0.021. Furthermore, a significantly higher proportion of pharmacists were aware of the legal aspects of antibiotic dispensing compared to the assistants (χ^2^ (1, N = 265) = 8.55, *p* = 0.003) (Table 2). However, less than a quarter of the staff (22%; 58/259) correctly defined the term “antibiotic” 24% (49/205) pharmacists and 2% (9/54) of the pharmacy assistants. The correct responses provide to the ABR related items included, knowing that “resistant bacteria can be spread in health institutions and communities” (57% of pharmacists and 56% of assistants responded correctly) and that “dispensing antibiotics shorter than normal course by a pharmacist is one of the causes of ABR” (34% of pharmacists, 29% of assistants). Similarly, items measuring misuse of antibiotics were also correctly responded by about half or less than half of the staff; these items included, “viral diseases can be treated with antibiotics” (56% of pharmacists, 53% of assistants), “Antibiotics can be used as a preventive measure to fight against future microbial attacks” (38% of pharmacists, 33% of assistants), and correct knowledge about treating acute sore throat (32% of pharmacists, 24% of assistants) and acute diarrhoea (57% of pharmacists, 55% of assistants) with antibiotics were also low.

**Table 2:** Knowledge scores of pharmacy staff.

	All the Respondents	Pharmacy assistants Mean (±SD)	Pharmacists Mean (±SD)	t-test *p* value
Overall knowledge (n = 265) (Possible score range—0–34)	26.07 (3.86)	24.96 (3.86)	26.36 (3.82)	.017
Knowledge about Antibiotic (n = 263) (Possible score range—0–3)	1.89 (.69)	1.87 (.64)	1.90 (.70)	.801
Knowledge about ABR (n = 264) (Possible score range—0–12)	9.17 (1.67)	8.64 (1.97)	9.32 (1.56)	.021
Knowledge about antibiotic use/ misuse (n = 264) (Possible score range—0–14)	10.42 (2.06)	10.05 (2.10)	10.51 (2.04)	.144
Knowledge about legal aspect of antibiotic use (n = 265)				0.003^b^
Low knowledge	127 (49.90)^a^	36 (65.5)^a^	91 (43.3)^a^	
High knowledge	138 (52.10)^a^	19 (34.5)^a^	119 (56.70)^a^	

^a^ Proportion and percentage;

^b^Chi-square test *P* value.

### Self-reported antibiotic dispensing practice

As Table 3 illustrates, one in every three-pharmacy staff reported that they dispensed antibiotics without a prescription on patient request and the proportion of such dispensing practice increased to about half when the patient was known to them. Overall, 30% of the staff reported that they dispensed antibiotics without a prescription when considering all of the infections assessed, including, acute sore throat, common cold, acute diarrhoea, wound infection or uncomplicated UTI in the week prior to completing the survey. The highest reported antibiotic dispensing was for wound infections (21% of responding staff). The dispensing practice was not significantly different between pharmacists and pharmacy assistants (*p*>0.05) in general. However, a significantly higher proportion of the pharmacy assistants reported having dispensed antibiotics without a prescription compared to the pharmacists in the past seven days for sore throat (χ^2^ (1, N = 253) = 4.20, *p* = 0.040).

**Table 3:** Self-reported antibiotic dispensing practice.

Dispensing practice items	Frequency (%), N = 265			
	All the Respondents	Non-Pharmacist	Pharmacist	χ^2^ *p* value
**Dispense antibiotics without a prescription on patient demand**				0.805
Never	179 (67.5)	36 (66.7)	143 (68.4)	
Yes	84 (31.7)	18 (33.3)	66 (31.6)	
MD	2 (.8)			
**If I know the patient, I dispense antibiotics without a prescription on the patient’s request**				0.055
Never	147 (55.5)	32 (59.3)	115 (54.8)	
Yes	117 (44.2)	22 (40.7)	95 (45.2)	
MD	1 (.4)			
**I give antibiotics without prescription for adult patients with minor ailments caused by viral infection**				0.077
Never	233 (87.9)	51 (96.2)	182 (87.9)	
Yes	27 (10.2)	2 (3.8)	25 (12.1)	
MD	5 (1.9)			
**Children who have viral infections, I dispense antibiotics without a prescription**				0.127^a^
Never	247 (93.2)	53 (100)	194 (94.6)	
Yes	11 (4.2)	0	11 (5.4)	
MD	7 (2.6)			
**I give antibiotics without a prescription for adult patients with minor ailments caused by bacterial infections**				0.640
Never	216 (81.5)	43 (79.6)	173 (82.4)	
Yes	48 (18.1)	11 (20.4)	37 (17.6)	
MD	1 (.4)			
**Children who have bacterial infections, I dispense antibiotics without a prescription**				0.555^a^
Never	245 (92.5)	49 (90.7)	196 (93.3)	
Yes	19 (7.2)	5 (9.3)	14 (6.7)	
MD	1 (.4)			
**Proportion of dispensed antibiotics without a prescription in the last week for following minor infections**				
**Acute sore throat**				0.040
Never (0%)	217 (81.9)	40 (76.9)	177 (88.1)	
Yes	36 (13.6)	12 (23.1)	24 (11.9)	
MD	12 (4.5)			
**Common cold and cough**				0.050
Never (0%)	214 (80.8)	38 (74.5)	176 (85.9)	
Yes	42 (15.8)	13 (25.5)	29 (14.1)	
MD	9 (3.4)			
**Wound infection**				0.940
Never (0%)	199 (75.1)	40 (78.4)	159 (77.9)	
Yes	56 (21.1)	11 (21.6)	45 (22.1)	
MD	10 (3.8)			
**Urinary tract infections**				1.000^a^
Never (0%)	231 (87.2)	47 (92.2)	184 (90.6)	
Yes	23 (8.7)	4 (7.8)	19 (9.4)	
MD	11 (4.2)			
**Diarrhoea**				0.190
Never (0%)	227 (85.7)	43 (84.3)	184 (90.6)	
Yes	27 (10.2)	8 (15.7)	19 (9.4)	
MD	11 (4.2)			
**Dispensed antibiotic without a prescription in the last week for any of minor infections**				0.753
Never (0%)	179 (67.5)	35 (67.3)	144 (69.6)	
Yes	80 (30.2)	17 (32.7)	63 (30.4)	
MD	6 (2.3)			

MD–Missing data;

^a^ Fisher’s exact test.

### Impact of community pharmacists’ knowledge, other socio-demographic and professional characteristics on antibiotic dispensing without a prescription

The pharmacists’ knowledge about ABR significantly reduced the likelihood of dispensing antibiotics without a prescription for viral infections in adults (Adj. OR = .73, 95% CI: .55-.96; *p* = .027) and children (Adj. OR = .55, 95% CI: .38-.80; *p* = .002). Knowledge of the legal aspects of antibiotic dispensing also significantly decreased the likelihood of non-prescription antibiotic dispensing for unspecified patients (Adj. OR = .41, 95% CI: .21-.79; *p* = .008), the patients known to the pharmacist (Adj. OR = .29, 95% CI: .16 -.55; *p* < .001) requesting an antibiotic and adults with bacterial infections (Adj. OR = .45, 95% CI: .20-.99; *p* = .047) (Table 4).

Pharmacist employees were less likely to dispense antibiotics without a prescription for a direct patient request compared to owner pharmacists (Adj. OR = .43, 95% CI: .22 - .85; *p* = .015) (Table 4). All other demographic characteristics, such as gender, geographical area, years of community pharmacy experience and employment status, once adjusted in the regression model, were not significantly associated with non-prescription antibiotic dispensing practice.

**Table 4:** Impact of pharmacists’ knowledge and socio-demographic on antibiotic dispensing practice in general.

Predictors	Dispensing antibiotic without a prescription Adj.OR (95% CI) *p*					
	On patient’s direct request	On known patient’s direct request	For adult with minor viral infection	For children with minor viral infections	For adult with minor bacterial infections	For children with minor bacterial infections
Knowledge about ABR	1.12(.89, 1.40)^NS^	1.24(.99, 1.53)^NS^	.73(.55, .96)*	.55(.38, .80)**	.92(.72, 1.18)^NS^	1.00(.66 1.50)^NS^
Knowledge about antibiotic use/ misuse	1.03(.78, 1.21)^NS^	.95(.82, 1.11)^NS^	1.02(.82, 1.28)^NS^	1.02(.73, 1.41)^NS^	.89(.73, 1.08)^NS^	.69(.49, .96)*
Knowledge about legal aspect of antibiotic use						
Low	1	1	1	1	1	1
High	.41(.21, .79)**	.29 (.16, .55)***	.80(.32, 2.01)^NS^	1.81(.44, 7.44)^NS^	.45(.20, .99)*	.37(.09, 1.47)^NS^
Age	.98(.94, 1.02)^NS^	.96(.92, .99)*	.94(.88, 1.01)^NS^	1.00(.92, 1.09)^NS^	.93 (.87, .98)*	1.08(1.01, 1.15)*
**Employment Type**						
Owner	1	1	1	1	1	1
Employee	.43 (.22, .85)*	1.08(.58, 2.04)	.70(.27, 1.82)^NS^	.88(.21, 3.75)^NS^	.69(.31, 1.57)^NS^	.41(.11, 1.54)^NS^

*p* value:

***<0.001,

**<0.01,

*<0.05;

^NS^ not significant.

Other predictors adjusted in the model: Knowledge about antibiotic, gender, geographical area, years of community pharmacy experience and employment status.

As Table 5 describes, Pharmacists’ knowledge about antibiotic use/ misuse significantly reduced the likelihood of dispensing antibiotics without a prescription for common cold (Adj. OR = .75, 95% CI: .60 - .94; *p* = .011) and acute diarrhoea (Adj. OR = .76, 95% CI: .58 - .99; *p* = .047). However, other knowledge items and demographic characteristics such as geographical location of the pharmacy, years of community pharmacy experience, employment status and employment type, once adjusted in the regression model were not significantly associated with antibiotic dispensing practice without a prescription for the minor infections.

**Table 5:** Impact of pharmacists’ knowledge and socio-demographic on dispensing antibiotic past week for minor infections.

Predictors	Dispensing antibiotic without a prescription Adj. OR (95% CI) *p*				
	Acute sore throat	Common cold	Wound infections	UTI	Diarrhoea
Knowledge about AB	.81(.43, 1.54)^NS^	1.19(.65, 2.20)^NS^	.73(.44, 1.21)^NS^	.55(.26, 1.16)^NS^	.99(.47, 2.10)^NS^
Knowledge about ABR	.83(.61, 1.11)^NS^	1.01(.78, 1.43)^NS^	1.01(.79, 1.29)^NS^	1.09(.75, 1.82)^NS^	.94(.66, 1.34)^NS^
Knowledge about antibiotic use/ misuse	.81(.64, 1.02)^NS^	.75(.60, .94)*	.89(.74, 1.07)^NS^	.86(.66, 1.13)^NS^	.76(.58, .99)*
Knowledge about legal aspect of antibiotic use					
Low	1	1	1	1	1
High	1.23(.46, 3.33)^NS^	.94(.38, 2.29)^NS^	.63(.30, 1.32)^NS^	.49(.17, 1.45)^NS^	.93(.31, 2.81)^NS^
Age	.95(.89, 1.02)^NS^	.94(.88, 1.00)^NS^	.95(.90, 1.0)^NS^	.97(.91, 1.31)^NS^	.96(.89, 1.03)^NS^

*P* value:

*<0.05;

^NS^ not significant.

Other predictors adjusted in the model: gender, geographical area, years of community pharmacy experience, employment status and employment type.

## Discussion

To the best of our knowledge, this is the first nationwide study from Sri Lanka evaluating the self-reported antibiotic dispensing practice of community pharmacy staff (pharmacists and pharmacy assistants) and the impact of pharmacists’ knowledge and professional and demographic characteristics on antibiotic dispensing.

This study, demonstrated that whilst pharmacists showed a greater knowledge about ABR and legal aspects of antibiotic dispensing than the pharmacy assistants, they had an overall poor knowledge of antibiotics (definition), appropriate antibiotic use and antibiotic resistance. This poor knowledge was reflected in the self-reported behaviour, with a large proportion of participants providing antibiotics to patients on request, and inappropriately in situations where patients may have had viral infections. The study findings also demonstrated that pharmacists who reported knowing about the legal requirements for supplying antibiotics with a prescription and who were knowledgeable about ABR and the use/ misuse of antibiotics, were less likely to dispense antibiotics without a prescription.

Our findings regarding poor knowledge about antibiotics are similar to findings from other studies conducted in LMICs [12–14, 22]. Apisarnthanarak *et*.*al*, in 2008, found that around 24% of Thai pharmacists had inadequate knowledge about antibiotics [14]. A study conducted in Indian rural pharmacies found that half of the employees could correctly define antibiotics and two-thirds of them had not heard about ABR [12]. The knowledge gap we found among the pharmacists can be explained by the limited professional and clinical training of pharmacists in Sri Lanka, particularly as a majority of the community pharmacists in Sri Lanka are apprentice pharmacists without a formal tertiary education or clinical training [23]. Thus, limited knowledge about antibiotics, their use/ misuse, as well as ABR could be a large factor influencing illegal and inappropriate supply of antibiotics without prescription to patients with minor infections presenting at community pharmacies. This educational gap presents an opportunity for strategies to be developed to address the need for further education and which can be easily addressed in the short term, both at all types of pharmacy programs and at the practising pharmacy level through the delivery of targeted continuing educational programs. Educating and upskilling pharmacists will have a long term impact on reducing the illegal and inappropriate supply of antibiotics to the public and will also ensure that pharmacists take up the role of educating not only their own staff in the pharmacy, but also the public about appropriate use of antibiotics. Overall, addressing this gap, will meet long term objectives of reduced antibiotic misuse and inappropriate use, and contribute to reduced antibiotic resistance nationally and globally [6, 24, 25]. Specifically, as Sri Lanka is one of the tourist destinations, millions of travellers visit Sri Lanka every year from all over the world and studies show that resistance pathogens can be transmitted globally with international travellers [25].

Targeted education of practising pharmacists and other staff may also specifically address other issues highlighted in this study; for example, provision of antibiotics more readily to patients known to the pharmacy staff. Familiarity with the patient appeared to be a reason why antibiotics were more likely to be provided without a prescription, with no significant difference observed between pharmacists and assistants. These findings are consistent with similar studies conducted to evaluate the inappropriate dispensing practice in LMICs [26, 27]. It is possible that pharmacy staff feel that they have a greater obligation to supply antibiotics to patients they know. It is also possible that they may feel that they are more familiar with the patients’ health and medical conditions and therefore are more comfortable in providing antibiotics without a prescription. Equally, the familiarity may also allow increased opportunities for monitoring the health of the patient and evaluating the impact of the short-term supply of an antibiotic.

In addition to inadequate clinical experience and knowledge about antibiotic dispensing contributing to the illegal and inappropriate antibiotic supply reported in this study, there may be other reasons explaining the behaviour of the pharmacists and pharmacy assistants. For example, pressure from the public to supply an antibiotic, the profit motive, disregard for their legal obligations related to antibiotic supply, lax law enforcement, lack of awareness about existing antibiotic dispensing laws and fear of losing customers [13].

However, our present study also revealed that having pharmacists with a better knowledge of ABR and use/ misuse could reduce antibiotic supply without prescription for viral infections (appropriate practice); and better knowledge related to dispensing regulations may reduce antibiotics supply without a prescription (illegal supply) to both known and unknown patients and for bacterial infections. Recent literature from other countries has also found similar relationships [13, 14, 28]. Therefore, in addition to continuous education for pharmacists, revising the curricula of pharmacy programs and strict enforcement of antibiotic dispensing related regulations in the country should also be established.

However, the control of ABR cannot be the sole responsibility of health professionals and scientists. The public and other stakeholders have a major role to play as well. Public awareness should be encouraged to minimise patient demand for antibiotics and optimise appropriate use. A survey of parents of school children in Italy found that about 10% and 21% correctly knew the definition of antibiotics and ABR, respectively, and one in every third person surveyed was willing to self-medicate with an antibiotic [29]. Internet and social media have become popular among the public as a source of antibiotic related information [30]. Therefore, these sources could also be considered as one of the key tools for public education on appropriate use of antibiotics.

The government should consider ABR as a major public health issue. Policies and regulations should be put in place to enforce appropriate access, minimise the public’s demand from health professionals and reduce inappropriate use of antibiotics. The media professionals should also be adequately trained on how to convey medical and scientific information in lay language to inform the populations on practices that promote the appropriate use of antibiotics. The empirical evidence may facilitate the development of educational and behavioural interventions, dispensing guides, and strengthening the policies which can be undertaken to combat against this public health issue.

### Limitations

Like most surveys, our study also has some limitations. There is a possibility of social desirability bias on the antibiotic dispensing practice-related issues, where the respondents may give more favourable responses about their antibiotic dispensing practice [13]. However, to address this limitation and assess the validity of the findings, two separate pseudo-patient visits to the same pharmacies directly requesting an antibiotic product (61%) [10] and presenting symptoms (41%) [11] were conducted. The comparison of the three methods will be the focus of a future publication.

Approximately 20–30 minutes was taken to complete the questionnaire, which could have impacted the responses through survey fatigue [31].

Another possible limitation of this study is non response bias caused by those who did not respond. However, in our study, the response rate was very high (72%), when compared to similar research [32] with responses from all the different types of pharmacies in Sri Lanka. The results of this study may only be applicable in environments where the legislative framework regarding antibiotic supply and pharmacists’ qualifications are comparable with Sri Lankan context.

## Conclusions

Despite Sri Lankan law prohibiting provision of antibiotics to the public without a prescription, this study revealed that pharmacy staff continue to provide antibiotics without a prescription. Pharmacists did not report an overall lower provision rate. The illegal and inappropriate antibiotics supply was associated with lower levels of pharmacists’ legal and clinical knowledge about antibiotics.

There is room for improvement in community pharmacy staff’s basic knowledge about antibiotics, antibiotic resistance, antibiotic use and misuse. It is important that the government strictly enforces the law regarding antibiotics supply. Furthermore, standards should be implemented and adopted on the appropriate provision and supply of antibiotics. Educational programmes and interventions should be developed and implemented to ensure that both newly qualifying pharmacists as well as currently practicing pharmacists in the community settings are well-informed about antibiotics, appropriate use and antibiotic resistance, as well as the legal requirements for supply.

## Supporting information

S1 Appendix Questionnaire. (PDF)
