# Supplementary material for: A cross-sectional national survey of community pharmacy staff: Knowledge and antibiotic provision
Source: PLoS One. 2019 Apr 25;14(4):e0215484. doi: 10.1371/journal.pone.0215484 (PMC6483176; doi:10.1371/journal.pone.0215484)
Supplement: S1 Appendix — (PDF) [file pone.0215484.s001.pdf]

Community Pharmacists' or Pharmacy Assistants' Knowledge & Practices Related to Dispensing Antibiotics in Sri Lanka

INSTRUCTIONS

Section 1: Knowledge Related to Antibiotics (K1-K34)
*For each statement you need to tick **one** relevant box only.*

Section 2: Antibiotic Dispensing Practice (P1-P7)
***P1-P6**: In this section, the statements ask you to indicate the practice related to antibiotics dispensing by selecting one of following options; "**never," "some of the time," "half of the time," "Most of the time," "Always.**"*

P7**: In this section, the statements ask you to indicate proportion of your practice such as **0%, 25%, 50%, 75% and 100% (where 0% reflects "never" and 100% means "always") by ticking the appropriate one box only.

Reference No: ………………………… **District: ………………………**

Name of the research Assistant:...

(Please answer all questions and statements)

Section 1: Knowledge related to antibiotics

*For each statement you need to tick **one** box only*

No	Statement	Yes	No	Unsure (US)
K1	An antibiotic is any agent used to kill or inhibit the growth of microorganisms (bacteria, fungus, virus, parasites).	□Yes	□No	□US
K2	Patient self-medication of antibiotics is one of the causes of antibiotic resistance.	□Yes	□No	□US
K3	Antibiotics are effective medicines that treat many infections.	□Yes	□No	□US
K4	Inappropriate use of antibiotics can lead to ineffective treatment.	□Yes	□No	□US
K5	Fever can be reduced directly with antibiotics.	□Yes	□No	□US
K6	If taken too often, antibiotics are less likely to work in the future.	□Yes	□No	□US
K7	Common cold and cough should always be treated with antibiotics.	□Yes	□No	□US
K8	Viral diseases can be treated with antibiotics.	□Yes	□No	□US
K9	Diphenhydramine is an antibiotic used in treating upper respiratory tract infections.	□Yes	□No	□US
K10	Antibiotics can be used as a preventive measure to fight against future microbial attacks.	□Yes	□No	□US
K11	Acute sore throat can be treated with antibiotics.	□Yes	□No	□US
K12	Keeping leftover antibiotics from a previous course to use next time for the same infection is a good practice.	□Yes	□No	□US
K13	Bacterial infections can be treated with antibiotics.	□Yes	□No	□US
K14	Wound infection can be treated with antibiotics.	□Yes	□No	□US
K15	Antibiotic resistance is an important and serious public health issue facing the world.	□Yes	□No	□US
K16	Patients can stop taking antibiotics when their symptoms improve.	□Yes	□No	□US
K17	Acute diarrhoea can be treated with antibiotics.	□Yes	□No	□US
K18	Dispensing antibiotics without a prescription is not a problem.	□Yes	□No	□US
K19	Dispensing antibiotics without a prescription contributes to inappropriate use of antibiotics by patients.	□Yes	□No	□US
K20	Inappropriate use of antibiotics increases the emergence of bacterial resistance to antibiotics.	□Yes	□No	□US
K21	Dispensing antibiotics without a prescription will lead to development of antibiotic resistance.	□Yes	□No	□US
K22	Resistant bacteria cannot be spread in healthcare institutions and communities.	□Yes	□No	□US
K23	Urinary tract infections can be treated with antibiotics.	□Yes	□No	□US
K24	Skipping one or two doses of an antibiotic does not contribute to the development of antibiotic resistance.	□Yes	□No	□US
K25	Lack of patient adherence to prescribed antibiotic treatment regimens is one of the main causes of antibiotic resistance.	□Yes	□No	□US
K26	I am not aware of the rules and regulations about dispensing antibiotics (with or without a prescription) in Sri Lanka.	□Yes	□No	□US
K27	Early cessation of an antibiotic course is one of the causes of antibiotic resistance.	□Yes	□No	□US
K28	Use of antibiotics greater than normal prescribed course is one of the causes of antibiotic resistance.	□Yes	□No	□US
K29	The occurrence of antibiotic resistance is mainly a problem in hospital settings.	□Yes	□No	□US

Section 1: Knowledge related to antibiotics

This section is about your knowledge of antibiotics. Please tick the relevant box in response to the statements.

For each statement you need to tick relevant ***one*** *box only*

No	Statement	Yes	No	Unsure (US)
K30	In Sri Lanka, antibiotics are categorised under schedule II- group B drugs (Prescription drugs).	□Yes	□No	□US
K31	An antibiotic is any agent used to kill or inhibit the growth of bacteria.	□Yes	□No	□US
K32	Pharmacists can legally dispense antibiotics without a prescription in Sri Lanka.	□Yes	□No	□US
K33	Dispensing antibiotic shorter than normal course by pharmacists is one of the main causes of antibiotic resistance.	□Yes	□No	□US
K34	Pharmacists can be penalised for dispensing antibiotics without a prescription.	□Yes	□No	□US

Section 2: Antibiotic Dispensing Practice

Please tell us how frequently you perform the followings, by ticking the relevant box below for each statement.

For ***each*** *statement you need to tick* ***one*** *box only*

No.	Statement	Never (1)	Some of the time (2)	Half of the time (3)	Most of the time (4)	Always (5)
P1	I dispense antibiotics without a prescription if a patient requests an antibiotic.	□ 1	□ 2	□ 3	□ 4	□ 5
P2	I give antibiotics without prescription for adult patients with minor ailments caused by viral infections.	□ 1	□ 2	□ 3	□ 4	□ 5
P3	Children who have viral infections, I dispense antibiotics without a prescription.	□ 1	□ 2	□ 3	□ 4	□ 5
P4	Children who have bacterial infections, I dispense antibiotics without a prescription.	□ 1	□ 2	□ 3	□ 4	□ 5
P5	If I know the patient, I dispense antibiotics without a prescription on the patient's request.	□ 1	□ 2	□ 3	□ 4	□ 5
P6	I give antibiotics without a prescription for adult patients with minor ailments caused by bacterial infections.	□ 1	□ 2	□ 3	□ 4	□ 5

Section 2: Antibiotic Dispensing Practice
Please tell us how frequently you perform the followings, by ticking the relevant box below. The values range from 0 to 100%. If you NEVER perform the behaviour, you can tick 0%. If you ALWAYS perform the behaviour, you can tick 100%.

For each statement you need to tick relevant ***one*** *box only*

No.	Statement	Never (0%) 1	25% 2	50% 3	75% 4	Always (100%) 5	Don't know (DK)
P7	**Please let us know for the following condition/s the proportion of antibiotics you have dispensed without prescription in the last week.**						
	1. Acute sore throat.	□ 1	□ 2	□ 3	□ 4	□ 5	□ DK
	2. Common cold and cough.	□ 1	□ 2	□ 3	□ 4	□ 5	□ DK
	3. Wound infection.	□ 1	□ 2	□ 3	□ 4	□ 5	□ DK
	4. Urinary tract infections.	□ 1	□ 2	□ 3	□ 4	□ 5	□ DK
	5. Diarrhoea.	□ 1	□ 2	□ 3	□ 4	□ 5	□ DK

Section 3: Socio-Demographic Data

	Socio-Demographic Characteristics	Responses
1.	How many years of experience have you had as a community pharmacist?	
2.	How many registered pharmacists in total, work in your pharmacy?	
3.	How many registered pharmacists work at any one time in your pharmacy?	
4.	What is your age (Years)?	
5.	Please state the area where your pharmacy is?	☐ Municipal council<br>☐ Urban council<br>☐ Town council
6.	Please indicate your gender	☐ Male<br>☐ Female
7.	What is your level of Pharmacy education?	☐ Efficiency Pharmacy<br>☐ Proficiency Pharmacy<br>☐ Degree (B.Pharm/ BSc Pharmacy)<br>☐ Other specify.................................
8.	What is the type of pharmacy that you work in?	☐ Rajya Osusala<br>☐ Private pharmacy chain (groups of pharmacies)<br>☐ Single private pharmacy<br>☐ Pharmacy at a private hospital<br>☐ Other specify................................
9.	What is your employment type?	☐ Pharmacy owner<br>☐ Employee
10.	What is your employment status?	☐ Full time<br>☐ Part time

ශ්‍රී ලංකාවේ ප්‍රතිජීවක ලබා දීම සම්බන්ධව ප්‍රජා ඖෂධවේදීන්ගේ දැනුම සහ වෘත්තීය නියැලුම

(ප්‍රජා ඖෂධවේදීන්ගේ ප්‍රශ්නාවලිය)

උපදෙස්

1 කොටස: ප්‍රතිජීවක සම්බන්ධව දැනුම (K1-K34)
*සෑම වගන්තියක් සඳහාම උචිත **එක්** කොටුවක් පමණක් සළකුණු කරන්න.*

2 කොටස: ප්‍රතිජීවක ලබා දීමේ වෘත්තීය නියැලුම (P1-P7)

P1-P6: *මෙම කොටසේදී, වගන්ති සඳහා ප්‍රතිජීවක නිකුත් කිරීමේ ස්වභාවය "**කවදාවත් නැත**", "**ඉදහිට**", "**භාග විට**", "**බොහෝ විට**" සහ "**සෑමවිටම**" ලෙස එක් නියමිත කොටුවක පමණක් සළකුණු කරන්න.*

P7: *මෙම කොටසේදී, වගන්ති සඳහා ඔබේ වෘත්තීය නියැලුමේ ස්වභාවය ප්‍රමාණයක් ලෙස **0%, 25%, 50%, 75% සහ 100% (0% යනු "කවදාවත් නැත" සහ 100% යනු "සෑමවිටම" වේ)** ලෙස එක් නියමිත කොටුවක පමණක් සළකුණු කරන්න.*

යොමු අංකය: .... **දිස්ත්‍රික්කය: ....**

Name of the Research Assistant…………………………………………………………

(කරුණාකර සෑම ප්‍රශ්ණයකටම සහ වගන්තියකටම පිළිතුරු සපයන්න)

1 කොටස: ප්‍රතිජීවක සම්බන්ධව දැනුම

මෙම කොටස ප්‍රතිජීවක සම්බන්ධව ඔබේ දැනුම පිළිබදවයි. කරුණාකර වගන්ති සඳහා පිළිතුරු ලෙස අදාළ කොටුව සළකුණු කරන්න.

*සෑම වගන්තියක් සඳහාම උචිත **එක්** කොටුවක් පමණක් සළකුණු කරන්න*

අංකය	වගන්තිය	ඔව්	නැත	අවිනිශ්චිතයි
K1	ප්‍රතිජීවක ලෙස හදුන්වන්නේ ක්ෂුද්‍ර ජීවීන් (බැක්ටීරියා, දිලීර, වෛරස්, පරපෝෂිතයන්) විනාශ කිරීමට හෝ වර්ධනය නිෂේධ කිරීමට භාවිත කරන	□ ඔව්	□ නැත	□ අවිනිශ්චිතයි
K2	රෝගීන් වෛද්‍ය නිර්දේශ නොමැතිව ප්‍රතිජීවක භාවිතා කිරීම ප්‍රතිජීවක ප්‍රතිරෝධය (Antibiotic resistance) හටගැනීමට බලපාන හේතු වලින් එකකි.	□ ඔව්	□ නැත	□ අවිනිශ්චිතයි
K3	ප්‍රතිජීවක යනු බොහෝ ආසාදන වලට ප්‍රතිකාර කරන ඵලදායී ඖෂධයකි.	□ ඔව්	□ නැත	□ අවිනිශ්චිතයි
K4	ප්‍රතිජීවක නිසි පරිදි භාවිතා නොකිරීම, ප්‍රතිකාරය නිශ්ඵල කිරීමට මග [illegible]	□ ඔව්	□ නැත	□ අවිනිශ්චිතයි
K5	ප්‍රතිජීවක මඟින් උණ සෘජුවම අඩු කල හැක.	□ ඔව්	□ නැත	□ අවිනිශ්චිතයි
K6	නිතර නිතර භාවිතකල හොත් ප්‍රතිජීවක අනාගතයේදී ක්‍රියා කිරීමේ සම්භාවිතාව අඩු විය හැක.	□ ඔව්	□ නැත	□ අවිනිශ්චිතයි
K7	සාමාන්‍ය සෙම්ප්‍රතිශ්‍යාව සහ කැස්ස සැමවිටම ප්‍රතිජීවක මඟින් ප්‍රතිකාර	□ ඔව්	□ නැත	□ අවිනිශ්චිතයි
K8	වෛරස් රෝග වලට ප්‍රතිකාර කිරීමට ප්‍රතිජීවක භාවිත කල හැක.	□ ඔව්	□ නැත	□ අවිනිශ්චිතයි
K9	ඩයිෆීන්හයිඩ්‍රමින් (Diphenhydramine) යනු උත්තර ශ්වසන මාර්ගයේ ආසාදන වලට ප්‍රතිකාර කිරීමට භාවිත කරන ප්‍රතිජීවකයකි.	□ ඔව්	□ නැත	□ අවිනිශ්චිතයි
K10	අනාගත ක්ෂුද්‍රජීවී ආක්‍රමණ වලට විරුද්ධව සටන් කරන නිවාරණ ක්‍රියාමාර්ග ලෙස ප්‍රතිජීවක භාවිත කල හැක.	□ ඔව්	□ නැත	□ අවිනිශ්චිතයි
K11	ප්‍රතිජීවක මඟින් කෙටිකාලීන උගුර අමාරුව සුව කල හැක.	□ ඔව්	□ නැත	□ අවිනිශ්චිතයි
K12	ඉතිරි වන ප්‍රතිජීවක ප්‍රමාණය ඊළඟ අවස්ථාවේ ඇතිවන සමාන ආසාදනයකට ප්‍රතිකාරයක් සඳහා ළඟ තබා ගැනීම හොඳ ක්‍රියාමාර්ගයකි.	□ ඔව්	□ නැත	□ අවිනිශ්චිතයි
K13	බැක්ටීරියා ආසාදන ප්‍රතිජීවක මඟින් ප්‍රතිකාර කල හැක.	□ ඔව්	□ නැත	□ අවිනිශ්චිතයි
K14	තුවාල වලට ප්‍රතිකාර කිරීමට ප්‍රතිජීවක භාවිත කල හැක.	□ ඔව්	□ නැත	□ අවිනිශ්චිතයි
K15	ප්‍රතිජීවක ප්‍රතිරෝධය යනු ලෝකය මුහුණ දෙන වැදගත් සහ බරපතල පොදු සෞඛ්‍ය ගැටළුවකි.	□ ඔව්	□ නැත	□ අවිනිශ්චිතයි
K16	රෝග ලක්ෂණ යටපත් වන විට රෝගීන්ට ප්‍රතිජීවක ගැනීම නැවැත්විය	□ ඔව්	□ නැත	□ අවිනිශ්චිතයි
K17	කෙටිකාලීන විරේකයට ප්‍රතිජීවක මඟින් ප්‍රතිකාර කල හැක.	□ ඔව්	□ නැත	□ අවිනිශ්චිතයි
K18	වෛද්‍යවරයෙකු විසින් නිර්දේශිත ඖෂධ වට්ටෝරුවක් නොමැතිව ප්‍රතිජීවක ලබා දීම ගැටළුවක් නොවේ.	□ ඔව්	□ නැත	□ අවිනිශ්චිතයි
K19	බෙහෙත් වට්ටෝරුවක් (ප්‍රෙස්ක්‍රිප්ෂන්)නොමැතිව ප්‍රතිජීවක ලබා දීම රෝගීන් ප්‍රතිජීවක අනිසි භාවිතයට හේතු වේ.	□ ඔව්	□ නැත	□ අවිනිශ්චිතයි

1 කොටස: ප්‍රතිජීවක සම්බන්ධව දැනුම

මෙම කොටස ප්‍රතිජීවක සම්බන්ධව ඔබේ දැනුම පිළිබඳවයි. කරුණාකර වගන්ති සඳහා පිළිතුරු ලෙස අදාළ කොටුව සළකුණු කරන්න.

සෑම වගන්තියක් සඳහාම උචිත එක් කොටුවක් පමණක් සළකුණු කරන්න

අංකය	වගන්තිය	ඔව්	නැත	අවිනිශ්චිතයි
K20	ප්‍රතිජීවක අනිසි භාවිතය, ප්‍රතිජීවක වලට බැක්ටීරියා ප්‍රතිරෝධය මතු වීම වැඩි කරයි.	□ ඔව්	□ නැත	□ අවිනිශ්චිතයි
K21	බෙහෙත් වට්ටෝරුවක් (ප්‍රෙස්ක්‍රිප්ෂන්) නොමැතිව ප්‍රතිජීවක ලබා දීම ප්‍රතිජීවක ප්‍රතිරෝධය (Antibiotic resistance) වර්ධනයට හේතු වේ.	□ ඔව්	□ නැත	□ අවිනිශ්චිතයි
K22	ප්‍රතිරෝධක බැක්ටීරියා සෞඛ්‍ය ආයතන තුළ සහ මහජනයා තුළ පැතිරිය නොහැක.	□ ඔව්	□ නැත	□ අවිනිශ්චිතයි
K23	ප්‍රතිජීවක මඟින් මුත්‍ර මාර්ග ආසාදන සුව කල හැක.	□ ඔව්	□ නැත	□ අවිනිශ්චිතයි
K24	ප්‍රතිජීවක මාත්‍රා එකක් හෝ දෙකක් මඟ හැරීම ප්‍රතිජීවක ප්‍රතිරෝධය (Antibiotic resistance) වර්ධනයට දායක නොවේ.	□ ඔව්	□ නැත	□ අවිනිශ්චිතයි
K25	ප්‍රතිජීවක ප්‍රතිරෝධයට (Antibiotics resistance) බලපාන ප්‍රධාන හේතු වලින් එකක් වනුයේ වෛද්‍යවරයා විසින් නිර්දේශිත ප්‍රතිජීවක වට්ටෝරුව, රෝගියා ඒ අයුරින්ම භාවිතා නොකිරීමයි.	□ ඔව්	□ නැත	□ අවිනිශ්චිතයි
K26	ශ්‍රී ලංකාවේ, බෙහෙත් වට්ටෝරුවක් (ප්‍රෙස්ක්‍රිප්ෂන්) ඇතිව /නොමැතිව ප්‍රතිජීවක ලබා දීමේ නීති සහ රෙගුලාසි පිළිබඳ මම දැනුවත් නැත.	□ ඔව්	□ නැත	□ අවිනිශ්චිතයි
K27	රෝගීන් විසින් නිර්දේශිත ප්රතිජීවක ප්රමාණය අවසන් වීමට පෙර ප්‍රතිකාරය නැවැත්වීම ප්‍රතිජීවක ප්‍රතිරෝධය (Antibiotic resistance)	□ ඔව්	□ නැත	□ අවිනිශ්චිතයි
K28	ප්‍රතිජීවක අති භාවිතය ප්‍රතිජීවක ප්‍රතිරෝධය (Antibiotic resistance) හටගැනීමට බලපාන හේත වලින් එකකි.	□ ඔව්	□ නැත	□ අවිනිශ්චිතයි
K29	ප්‍රතිජීවක ප්‍රතිරෝධය ප්‍රධාන වශයෙන් රෝහල් පරිසර ආශ්‍රිත ගැටළුවකි.	□ ඔව්	□ නැත	□ අවිනිශ්චිතයි
K30	ශ්‍රී ලංකාව තුළ ප්‍රතිජීවක II උපලේඛනය- බී කාණ්ඩය (schedule II- group B) (Prescription drugs) ඖෂධ යටතේ වර්ග කරනු ලබයි.	□ ඔව්	□ නැත	□ අවිනිශ්චිතයි
K31	ප්‍රතිජීවක ලෙස හඳුන්වන්නේ බැක්ටීරියා විනාශ කිරීමට හෝ වර්ධනය නිෂේධ කිරීමට භාවිත කරන ඕනෑම කාරකයකටයි.	□ ඔව්	□ නැත	□ අවිනිශ්චිතයි
K32	ශ්‍රී ලංකාවේ, ඖෂධවේදීන්ට නීත්‍යානුකූලව බෙහෙත් වට්ටෝරුවක් (ප්‍රෙස්ක්‍රිප්ෂන්) නොමැතිව ප්‍රතිජීවක ලබා දිය හැක.	□ ඔව්	□ නැත	□ අවිනිශ්චිතයි
K33	ප්‍රතිජීවක ප්‍රතිරෝධයට බලපාන ප්‍රධාන හේතු වලින් එකක් වනුයේ ඖෂධවේදීන් විසින් ප්‍රතිජීවක නිර්දෙශ කල ප්‍රමාණයට වඩා අඩු ප්‍රමණයක්	□ ඔව්	□ නැත	□ අවිනිශ්චිතයි
K34	බෙහෙත් වට්ටෝරුවක් (ප්‍රෙස්ක්‍රිප්ෂන්) නොමැතිව ප්‍රතිජීවක ලබා දීම වෙනුවෙන් ඖෂධවේදීන්ට දඬුවම් පැමිණවිය හැක.	□ ඔව්	□ නැත	□ අවිනිශ්චිතයි

2 කොටස: ප්‍රතිජීවක ලබා දීමේ වෘත්තීය නියැලුම

පහත දෑ ඔබ කොපමණ ප්‍රමාණයකින් ඉටු කරන්නේද යන්න අදාළ කොටුවේ සළකුණු කර අපට පවසන්න.

*සෑම වගන්තියක් සඳහාම උචිත **එක්** කොටුවක් පමණක් සළකුණු කරන්න*

**අංකය**	**වගන්තිය**	**කවදාවත් නැත 1**	**ඉද හිට 2**	**භාග විට 3**	**බොහෝ විට 4**	***සැමවිටම* 5**
P1	රෝගියෙක් බෙහෙත් වට්ටෝරුවක් (ප්‍රෙස්ක්‍රිප්ෂන්) නොමැතිව ප්‍රතිජීවකයක් ඉල්ලයි නම් මම ඒ ප්‍රතිජීවකය ලබාදෙමි.	□ 1	□ 2	□ 3	□ 4	□ 5
P2	වෛරස් ආසාදන නිසා ඇති වන සුළු අසනීප සහිත වැඩිහිටි රෝගීන් සඳහා මම බෙහෙත් වට්ටෝරුවක් (ප්‍රෙස්ක්‍රිප්ෂන්) නොමැතිව ප්‍රතිජීවක ලබාදෙමි.	□ 1	□ 2	□ 3	□ 4	□ 5
P3	වෛරස් ආසාදන සහිත ළමයින්ට මම බෙහෙත් වට්ටෝරුවක් (ප්‍රෙස්ක්‍රිප්ෂන්) නොමැතිව ප්‍රතිජීවක ලබාදෙමි.	□ 1	□ 2	□ 3	□ 4	□ 5
P4	බැක්ටීරියා ආසාදන සහිත ළමයින්ට මම බෙහෙත් වට්ටෝරුවක් (ප්‍රෙස්ක්‍රිප්ෂන්) නොමැතිව ප්‍රතිජීවක ලබාදෙමි.	□ 1	□ 2	□ 3	□ 4	□ 5
P5	රෝගියාව දන්නා අවස්ථාවකදී, බෙහෙත් වට්ටෝරුවක් (ප්‍රෙස්ක්‍රිප්ෂන්) නොමැතිව රෝගියාගේ ඉල්ලීම මත මම ප්‍රතිජීවක ලබාදෙමි.	□ 1	□ 2	□ 3	□ 4	□ 5
P6	බැක්ටීරියා ආසාදන නිසා ඇති වන සුළු අසනීප සහිත වැඩිහිටි රෝගීන් සඳහා මම බෙහෙත් වට්ටෝරුවක් (ප්‍රෙස්ක්‍රිප්ෂන්) නොමැතිව ප්‍රතිජීවක ලබාදෙමි.	□ 1	□ 2	□ 3	□ 4	□ 5

2 කොටස: ප්‍රතිජීවක ලබා දීමේ වෘත්තීය නියැලුම

පහත දෑ ඔබ කොපමණ ප්‍රමාණයකින් ඉටු කරන්නේද යන්න අදාළ කොටුවේ සළකුණු කිරීමෙන් අපට පවසන්න.
මෙම කොටසේදී, වගන්ති සඳහා ඔබේ වෘත්තීය නියැලුමේ ස්වභාවය ප්‍රමාණයක් ලෙස 0% සිට 100% දක්වා ලකුණු කරනු ලැබේ. ඔබ කිසිදිනක සිදුකර නැතිනම්, 0% කොටුව සළකුණු කරන්න. ඔබ සැමවිටම සිදුකරයි නම් 100% ලෙස සළකුණු කරන්න.

සෑම වගන්තියක් සඳහාම උචිත එක් කොටුවක් පමණක් සළකුණු කරන්න

අංකය	වගන්තිය	කවදාවත් නැත 0% (1)	25% (2)	50% (3)	75% (4)	සැමවිටම 100% (5)	නොදනී (6)
P7	කරුණාකර <u>පසුගිය සතිය තුළ</u> පහත තත්ත්වයන් වලට බෙහෙත් වට්ටෝරුවක් <u>නොමැතිව</u> (ප්‍රෙස්ක්‍රිප්ෂන්) ඔබ ලබාදුන් ප්‍රතිජීවක වල ප්‍රමාණය ප්‍රතිශතයක් ලෙස අපට සඳහන් කරන්න.						
	1. කෙටිකාලීන උගුර අමාරුව	□ 1	□ 2	□ 3	□ 4	□ 5	□ 6
	2. සාමාන්‍ය සෙම්ප්‍රතිශ්‍යාව සහ කැස්ස	□ 1	□ 2	□ 3	□ 4	□ 5	□ 6
	3. තුවාල	□ 1	□ 2	□ 3	□ 4	□ 5	□ 6
	4. මුත්‍ර මාර්ග ආසාදන	□ 1	□ 2	□ 3	□ 4	□ 5	□ 6
	5. කෙටිකාලීන විරේකය	□ 1	□ 2	□ 3	□ 4	□ 5	□ 6

3 කොටස: ඔබ පිළිබද තොරතුරු

අංකය	ඔබ පිළිබද තොරතුරු	කරුණාකර ලියන්න
1	ප්‍රජා ඖෂධවේදියෙකු (Community pharmacist) ලෙස අත්දැකීම් තිබෙන අවුරුදු ගණන කීයද?	
2	ඔබේ ෆාමසියේ වැඩකරන ලියාපදිංචි ඖෂධවේදීන් ගණන කීය	
3	ඔබේ ෆාමසියේ ඕනෑම එක් අවස්ථාවක දී වැඩකරන ලියාපදිංචි ඖෂධවේදීන් ගණන කීයද?	
4	ඔබේ වයස කීයද?	
5	ඔබගේ ෆාමසිය අයත් වන්නේ කිනම් බල ප්‍රදේශයකටද?	☐ මහ නගර සභා<br>☐ නගර සභා<br>☐ ප්‍රාදේශීය සභා
6	ඔබේ ස්ත්‍රී පුරුෂ බාවය	☐ පිරිමි<br>☐ ගැහැනු
7	ඖෂධවිද්‍යාව පිළිබද ඔබේ අධ්‍යාපන මට්ටම කුමක්ද?	☐Efficiency Pharmacy ☐**O/L**<br>☐Proficiency Pharmacy ☐**A/L**<br>☐උපාධිය (B.Pharm/ BSc Pharmacy)<br>☐Pharmacy Trainee<br>☐වෙනත්(සඳහන් කරන්න)……………

8	ඔබ වැඩකරන ෆාමසිවර්ගය කුමක්ද?	▢රජය ඔසුසල<br>▢පුද්ගලික ෆාමසි දාමය (Private pharmacy chain)<br>▢තනි පුද්ගලික ඖෂධාගාරය<br>▢පෞද්ගලික රෝහලක ඇති ඖෂධාගාරය
9	ඔබේ සේවා නියුක්තියේ ආකාරය කුමක්ද?	▢අයිතිකරු<br>▢ෆාමසියේ සේවයේ නියුතු
10	ඔබේ රැකියා තත්ත්වය කුමක්ද?	▢ පූර්ණ කාලීන<br>▢ අර්ධ කාලීන

ඔබේ වටිනා කාලයට සහ ප්‍රතිචාර වලට ස්තූතියි.

இலங்கையிலுள்ள சமூக மருந்தகவியலாளர்களின் (community pharmacists) antibiotics தொடர்பான அறிவு (விளக்கம்) மற்றும் நடத்தைகள்.

மருந்தகவியலாளர்களுக்கான (Pharmacist) வினாக்கொத்து

அறிவுறுத்தல்கள்

பகுதி 1 Antibiotics பற்றிய அறிவு (விளக்கம்) (K1 - K34)
ஒவ்வொரு கூற்றிக்கும் உரிய **ஒரு** கூட்டில் மட்டும **"√"**என அடையாளமிடுக.

பகுதி 2 Antibiotics **வழங்குவதிலுள்ள நடத்தைகள் (P1 – P7)**

P1-P6: எந்த அளவுக்கு நீங்கள் கீழ்வரும் நடவடிக்கைகளில் ஈடுபடுகிறீர்கள் என்பதை உரிய கூட்டில் "√" அடையாளமிடுவதன் மூலம் தெரிவிக்கவும்.; **"ஒருபோதும்இல்லை," "சில நேரங்களில்," "அரைவாசி நேரங்களில்," "பெரும்பாலான நேரங்களில," "எப்போதும்."**

P7: இப்பகுதியிலுள்ள கூற்றுக்களை எந்தளவு நீங்கள் நடைமுறைப்படுத்துகிறீர்கள் என்பதை சதவீதத்தில் **0%, 25%, 50%, 75%, 100%** என்றவாறு உரிய **ஒரு** கூட்டில் மட்டும் **"√"** என அடையாளப்படுத்துக. **0% என்பது "ஒரு போதும் இல்லை" என்பதையும் 100% என்பது "எப்போதும்"** என்பதையும் குறிக்கிறது.

குறியீட்டு இலக்கம் : .... **மாவட்டம்** : ....

Name of the Research Assistant……………………………………………………

பகுதி - 1 : **Antibiotics பற்றிய அறிவு (விளக்கம்)**

தயவு செய்து கீழுள்ள கூற்றுக்களுக்குரிய தங்களின் கருத்துக்கு எதிரே “√” அடையாளமிடுக.

இல	கூற்று	ஆம் (1)	இல்லை (2)	சரியாகத் தெரியாது(3)
K1	Antibiotics என்பது நுண்ணங்கிகளை (பக்ரீறியா,பங்கஸ், ஒட்டுண்ணிகள்,வைரஸ்) கொல்லும்/அவற்றின் வளர்ச்சியை தடுக்கும் மருந்து ஆகும்.	□ 1	□ 2	□ 3
K2	Antibiotics மருந்துகளை நோயாளிகள் மருத்துவரின் பரிந்துரையின்றி தாமாக உள்ளெடுத்தல் antibiotic resistance ஏற்படுவதற்கு ஒரு காரணமாகும்.	□ 1	□ 2	□ 3
K3	Antibiotics பல்வேறு தொற்றுக்களிற்கு சிகிச்சையளிக்க பயன்படும் சிறந்த மருந்து ஆகும்.	□ 1	□ 2	□ 3
K4	முறையற்ற antibiotic பாவனை வினைத்திறனற்ற சிகிச்சை முறையை ஏற்படுத்த முடியும்.	□ 1	□ 2	□ 3
K5	Antibiotics மூலம் காய்ச்சலை நேரடியாக குறைக்க முடியும்.	□ 1	□ 2	□ 3
K6	Antibiotic களை அடிக்கடி பாவிப்பதனால் எதிர்காலத்தில் அவற்றின் செயற்றிறன் குறைவடையும்.	□ 1	□ 2	□ 3
K7	சாதாரண தடிமல், இருமல்களுக்கு சிகிச்சை அளிக்க antibiotics பாவிக்கப்பட வேண்டும்.	□ 1	□ 2	□ 3
K8	வைரஸ் நோய்களிற்கு சிகிச்சை அளிக்க antibiotics பயன்படுத்தலாம்.	□ 1	□ 2	□ 3
K9	Diphenhydramine மேற் சுவாசத்தொகுதியில் ஏற்படும் தொற்றுகளை குணப்படுத்த பயன்படும் ஒரு antibiotic ஆகும்	□ 1	□ 2	□ 3
K10	ஏதிர்காலங்களில் நுண்ணுயிர் தாக்கங்களைத்(microbial attacks) தடுக்க (as a prophylaxis) antibiotics பாவிக்க முடியும்.	□ 1	□ 2	□ 3
K11	தொண்டை அரிப்பிற்கு சிகிச்சை அளிக்க Antibioticslg; பயன்படுத்தலாம்.	□ 1	□ 2	□ 3
K12	முன்னர் பாவித்து மீதமுள்ள antibioticsl மீண்டும் அதே நோய் நிலைமை ஏற்பட்டால் பாவிப்பதற்காக வைத்திருத்தல் சிறந்த பயிற்சியாகும்.	□ 1	□ 2	□ 3
K13	பக்ரீறியா நோய்களிற்கு சிகிச்சை அளிக்க antibiotics களைப் பயன்படுத்தலாம்.	□ 1	□ 2	□ 3
K14	புண்களிற்கு சிகிச்சை அளிக்க antibiotics களைப் பயன்படுத்தலாம்.	□ 1	□ 2	□ 3
K15	Antibiotic resistance உலகம் எதிர் நோக்கும் முக்கியமானதும் பாரதுாரமானதுமான சமூக சுகாதார பிரச்சனையாகும்.	□ 1	□ 2	□ 3
K16	நோயாளர்கள், நோய் அறிகுறிகள் குறைவடையும் போது antibiotic உள்ளெடுப்பதை இடையில் நிறுத்தலாம்.	□ 1	□ 2	□ 3
K17	சாதாரண வயிற்றுப்போக்கு (acute diarrhoea) சிகிச்சை அளிக்க antibiotics களைப் பயன்படுத்தலாம்.	□ 1	□ 2	□ 3
K18	Antibiotic களை prescription இன்றி விநியோகிப்பது பிரச்சினையன்று	□ 1	□ 2	□ 3
K19	Antibiotics களை prescription இன்றி விநியோகிப்பது நோயாளர்களால் முறையற்ற antibiotics பாவனைக்கு பங்களிக்கும்	□ 1	□ 2	□ 3
K20	முறையற்ற antibiotics பாவனையானது antibiotic resistance ஏற்படுத்தலை அதிகரக்கும்.	□ 1	□ 2	□ 3
K21	Antibiotic களை prescription இன்றி விநியோகிப்பது antibiotic resistanceஐ ஏற்படுத்தும்.	□ 1	□ 2	□ 3
K22	Resistant bacterias சுகாதார நிறுவனங்களிலும் சமூகத்திலும் பரவாது	□ 1	□ 2	□ 3
K23	சிறுநீரகவழியில் ஏற்படும் தொற்றுக்களிற்கு சிகிச்சை அளிக்க antibiotic களைப் பயன்படுத்தலாம்.	□ 1	□ 2	□ 3
K24	ஒன்று அல்லது இரண்டு வேளைகளில் antibioticsஐ தவறவிடுதல் Antibiotic resistance எற்படுவதில் பங்களிப்புச் செய்யாது.	□ 1	□ 2	□ 3
K25	பரிந்துரைக்கப்பட்ட antibiotics களை நோயாளர்கள் சரியாகப் பயன்படுத்தாமை antibiotic resistance ஏற்படுவதற்கு ஒரு முக்கிய காரணமாகும்.	□ 1	□ 2	□ 3
K26	எனக்கு இலங்கையில் antibiotics விநியோகிப்பது தொடர்பில்காணப்படும் சட்டங்கள் மற்றும் நெறிமுறைகள் பற்றி தெரியாது	□ 1	□ 2	□ 3
K27	பரிந்துரைக்கப்பட்ட காலத்திற்கு முன்பாகவே antibiotics உள்ளெடுப்பதை நிறுத்தல் antibiotic resistance ஏற்படுவதற்கு ஒரு காரணமாகும்.	□ 1	□ 2	□ 3

பகுதி - 1 : **Antibiotics** பற்றிய அறிவு (விளக்கம்)

தயவு செய்து கீழுள்ள கூற்றுக்களுக்குரிய தங்களின் கருத்துக்கு எதிரே "√" அடையாளமிடுக.

இல	கூற்று	ஆம் (1)	இல்லை (2)	சரியாகத் தெரியாது(3)
K28	பரிந்துரைக்கப்பட்ட அளவுக்கு அதிகமாக antibiotics பயன்படுத்தப்படல் Antibiotics resistance ஏற்படுவதற்கு ஒரு காரணமாகும்	□ 1	□ 2	□ 3
K29	Antibiotics resistance ஏற்படுதல் வைத்தியசாலைகளில் பிரதானமான பிரச்சனையாகும்.	□ 1	□ 2	□ 3
K30	இலங்கையில் Antibioticsஆனது அட்டவணை-2 "குழு B"(schedule II-groupB) மருந்துகளில் உள்ளடக்கப்பட்டுள்ளது(Prescription drugs).	□ 1	□ 2	□ 3
K31	Antibiotics என்பது பக்ரீறியாக்களை கொல்லும்/அவற்றின் வளர்ச்சியை தடுக்கும் மருந்துகளாகும்.	□ 1	□ 2	□ 3
K32	இலங்கையில் pharmacists சட்டரீதியாக prescription இன்றி antibiotic களை விநியோகிக்க முடியும்.	□ 1	□ 2	□ 3
K33	பரிந்துரைக்கப்பட்ட அளவை விட குறைந்த அளவு antibiotic களை pharmacist களால் வழங்கப்படுதல் antibiotic resistance ஏற்படுவதற்கு ஒரு முக்கியமான காரணமாகும்	□ 1	□ 2	□ 3
K34	மருந்தகவியலாளர்கள் antibiotic மருந்துகளை prescription இன்றி விநியோகித்தல் தொடர்பில் தண்டனைக்குள்ளாக்கப்பட முடியும்	□ 1	□ 2	□ 3

பகுதி - 2 **Antibiotics** வழங்கலிலுள்ள நடைமுறைகள்

எந்த அளவுக்கு நீங்கள் கீழ்வரும் நடவடிக்கைகளில் ஈடுபடுகிறீர்கள் என்பதை உரிய கூட்டில் "√" அடையாளமிடுவதன் மூலம் தெரிவிக்கவும்.

1. ஒருபோதும்இல்லை (1)
2. சில நேரங்களில் (2)
3. அரைவாசி நேரங்களில் (Half of the time) (3)
4. பெரும்பாலான நேரங்களில் (4)
5. எப்போதும் (5)

உரிய ஒரு கூட்டில் மட்டும் √ அடையாளமிடுக.

இல	கூற்று	1	2	3	4	5
P1	நோயாளி கோரும் பட்சத்தில் நான் prescription இன்றி antibioticsஐ விநியோகிக்கின்றேன்.	□ 1	□ 2	□ 3	□ 4	□ 5
P2	வயதுவந்த (adult) நோயாளர்களிற்கு வைரஸ்களினால் ஏற்படும் சிறிய நோய்களிற்கு நான் prescription இன்றி antibioticsஐ வழங்குகிறேன்.	□ 1	□ 2	□ 3	□ 4	□ 5
P3	வைரஸ் தொற்றுள்ள சிறுவர்களிற்கு நான் prescription இன்றி antibioticsஐ வழங்குகிறேன்.	□ 1	□ 2	□ 3	□ 4	□ 5
P4	பக்ரீறியாத் தொற்றுள்ள சிறுவர்களிற்கு நான் prescription இன்றி antibioticsஐ வழங்குகிறேன்.	□ 1	□ 2	□ 3	□ 4	□ 5
P5	நோயாளி தெரிந்தவராக இருக்கும் பட்சத்தில் அவரின் கோரிக்கையின் அடிப்படையில் நான் prescription இன்றி antibiotics ஐ விநியோகிக்கின்றேன்.	□ 1	□ 2	□ 3	□ 4	□ 5
P6	வயதுவந்த(adult) நோயாளர்களிற்கு பக்ரீறியாக்களினால் ஏற்படும் சிறிய நோய்களிற்கு நான் prescription இன்றி antibiotics ஐ வழங்குகிறேன்.	□ 1	□ 2	□ 3	□ 4	□ 5

பகுதி - 2 **Antibiotics** வழங்கலிலுள்ள நடைமுறைகள்

எந்த அளவுக்கு நீங்கள் கீழ்வரும் நடவடிக்கைகளில் ஈடுபடுகிறீர்கள் என்பதை உரிய கூட்டில் √ அடையாளமிடுவதன் மூலம் தெரிவிக்கவும். இச்சதவீதம் 0%-100% க்கு உட்பட்டது. நீங்கள் ஒருபோதும் கீழ்வரும் நடவடிக்கையில் ஈடுபடாவிடின்(Never) 0% எனவும் எப்போதும் ஈடுபடுபவர்(Always) எனின் 100% எனவும் உரிய கூட்டில் √ அடையாளமிடுக.

**No.**	**Statement**	**Never (0%) 1**	**25% 2**	**50% 3**	**75% 4**	**Always (100%) 5**	**Don't know (DK)**
P7	கடந்த வாரத்தில் prescription இன்றி கீழ்வரும் நிலைமை/நிலைமைகளுக்கு antibiotics வழங்கப்பட்ட சதவீதத்தை குறிப்பிடுக.						
	1. தொண்டை அரிப்பு (Acute sore throat)	□ 1	□ 2	□ 3	□ 4	□ 5	□ DK
	2. சாதாரண இருமல் தடிமன் (Common cold and cough.)	□ 1	□ 2	□ 3	□ 4	□ 5	□ DK
	3. புண்கள் (Wound infection)	□ 1	□ 2	□ 3	□ 4	□ 5	□ DK
	4. சிறுநீர் வழியில் ஏற்படும் தொற்று (Urinary tract infections)	□ 1	□ 2	□ 3	□ 4	□ 5	□ DK
	5. சாதாரண வயிற்றுப்போக்கு (Acute diarrhoea)	□ 1	□ 2	□ 3	□ 4	□ 5	□ DK

பகுதி – 3 சமூக ஜனநாயக தரவுகள்
இத்தரவுகளை வழங்குவதில் பங்களிப்பு செய்பவர்களின் சுயவிபரம்.

	**Socio-Demographic Characteristics**	**Responses**
1	Community pharmacist ஆக எத்தனை வருடகால அனுபவம் உங்களுக்கு உண்டு?	
2	எத்தனை பதிவு செய்யப்பட்ட pharmacists உங்களுடைய pharmacyஇல் பணிபுரிகின்றனர்?	
3	எத்தனை பதிவு செய்யப்பட்ட pharmacists உங்களுடைய மருந்தகத்தில் ஏதாவது ஒரு குறித்த நேரத்தில் பணிபுரிகின்றனர்?	
4	உங்களுடைய வயது என்ன?	
5	உங்களுடைய pharmacy அமைந்துள்ள இடம் எவ்வகையானது?	☐ Municipal council<br>☐ Urban council ☐ Town council
6	உங்களுடைய பால்	☐ ஆண் ☐ பெண்
7	உங்களுடைய Pharmacy கல்வி தகைமை என்ன?	☐ Efficiency Pharmacy<br>☐ Proficiency Pharmacy<br>☐ Degree (B.Pharm/ BSc Pharmacy)<br>☐ O/L ☐ A/L<br>☐ Other specify.................................
8	நீங்கள் வேலை பார்க்கும் pharmacy எவ்வகையானது?	☐ Rajya Osusala<br>☐ Private pharmacy chain (groups of pharmacies)<br>☐ Single private pharmacy<br>☐ Pharmacy at a private hospital<br>☐ Other specify................................
9	தங்களின் தொழிலின் வகை?	☐ Pharmacy உரிமையாளர்<br>☐வேலைசெய்பவர் (Employee)
10	தங்களின் தொழில் நியமனத்தின் வகை?	☐ Full time<br>☐ Part time
